# Acupuncture for osteoporosis: a systematic review protocol

**DOI:** 10.1186/s13643-016-0330-5

**Published:** 2016-09-21

**Authors:** Taipin Guo, Xiao Chen, Xiangnong Wu, Exian Shan, Yaju Jin, Xiantao Tai, Zili Liu, Bowen Zhu, Kai Yuan, Zukun Chen

**Affiliations:** 1 School of Acupuncture-Tuina and Rehabilitation, Yunnan University of Traditional Chinese Medicine, Kunming, Yunnan Province China; 2 Xi’an Encephalopathy Hospital of Traditional Chinese Medicine, Xi’an, Shanxi Province China; 3 Acupuncture Department, the First Affiliated Hospital, Yunnan University of Traditional Chinese Medicine, Kunming, Yunnan Province China

## Abstract

**Background:**

Osteoporosis is a global high prevalence of chronic metabolic disease with serious disability-adjusted life years losing. Acupuncture is used to treat osteoporosis broadly in China and other countries although the evidence on effectiveness cannot give a certain answer. The aim of this systematic review protocol is to appraise the efficacy and safety of acupuncture for osteoporosis.

**Methods:**

A literature search of randomized controlled trials focusing on acupuncture for osteoporosis will be performed in the databases of Medline, Cochrane Library, Web of Science, EBASE, Springer, WHO International Clinical Trials Registry Platform (ICTRP), China National Knowledge Infrastructure (CNKI), Wan fang, Chinese Biomedical Literature Database (CBM), Chinese Scientific Journal Database (VIP), and other possible resources with a valid search strategy. Outcomes of pain, bone mineral density, fracture, mortality, improvement proportion, biochemical indicators, quality of life, adverse event, and other valid will be extracted and merged for quantitative analysis using Review Manager software (V.5.3.5) or descriptive analysis correspondingly.

**Discussion:**

This is the first systematic review to estimate the effect of acupuncture on osteoporosis, and the result may provide evidence to clinical doctor.

**Systematic review registration:**

PROSPERO CRD42016037829

## Introduction

### Description of the condition

Osteoporosis is a chronic metabolic disease characterized by mass loss, microstructure damage, brittleness increases, strength decline, and fracture tendency of the bone. Osteoporosis can attack different gender and age, especially in postmenopausal woman and elderly male. The prevalence of osteoporosis is in a worldwide growth, such as 10.3 % in US older adults with 10.2 million people [1], 13.0 % in China mainland at the population of about 69.44 million [2, 3], and 6.6 and 22.1 % in men and women aged 50 years or more, and 5.5 % in the general population in the 27 countries of the European Union with 27.5 million suffering osteoporosis [4]. Those numbers are increasing accompanying aging population growth. Low back pain or systemic bone pain, deformation of spinal column, and fragility fracture are the main complaints of osteoporosis patient, which markedly accelerate the illness burden, as well as the personal and social financial expense. Based on the burden disease of disability-adjusted life years (DALY), osteoporosis fracture is ranked sixth, more than hypertension [5]. DALY losses are also considerable in postmenopausal osteoporosis [6]. The cost of incident and prior fragility fractures is 37 billion Euros in the 27 countries of the European in 2010, and it will rise by 25 % in 2015 owing to the tendency of aging population [4]. The aims of prevention and treatment of osteoporosis are to inhibit the rapid loss of bone mass, improve the quality and strength of bone, relieve the pain, and lower the risk of fracture, such as the basic therapy (e.g., combined calcium and vitamin D intake) and lifestyle changes (restriction of tobacco and alcohol intake, weight-bearing exercise, and avoidance of trip or fall hazards) and drugs (e.g., bisphosphonate, calcitonin, estrogen, parathyroid hormone, raloxifene, denosumab) [7]. However, the drugs could bring about many unavoidable side effects such as increase of the risk of cardiovascular disease, gastrointestinal reaction, rhinitis [7], and the basic therapy cannot effectively control the incidence. Complementary and alternative therapy such as acupuncture is applied more and more to treat osteoporosis [8, 9].

### Description of the intervention

Acupuncture therapy, with a history of more than 2000 years in China and some Asian countries, is to prevent and cure disease by the stimulation of specific acupoint according to Chinese meridians and “*Qi*” theory. There are different ways to stimulate the acupoint, such as needling, electro-acupuncture, transcutaneous electrical nerve stimulation (TENS), and moxibustion. Each acupoint has its own specific therapeutic functions, and the prescription of acupoint is formulated by different acupoints and special stimuli ways basing on ancient Chinese medicine theory [10].

### How the intervention might work?

The effect mechanism of osteoporosis by acupuncture has been not completely found so far. However, the accumulated evidences have shown that acupuncture could fortify bone strength by amending the mass, mineral density, and structure in osteoporosis model animal [11–14], which mainly activate the Wnt-β-catenin signaling pathway to influence bone formation and metabolism [15], as well as regulate the osteoprotegerin and receptor activator of nuclear factor-kB ligand (RANKL) [16].

### Why it is important to perform this review?

Osteoporosis is recognized as an aging and degenerative condition caused by insufficiency of kidney *qi* in line with theory of traditional Chinese medicine (TCM) in the ancient book of *Inner Canon of Huangdi* [17], and acupuncture is widely applied to treat it from 2000 years ago as well as herbs [18, 19]. In Australia, acupuncture is the second most popular complementary and alternative medicine after multivitamins for osteoporotic patient [20]. Nearly all TCM hospitals have employed acupuncture to treat osteoporosis in Chinese. The improvement of bone mineral density and analgesia are deemed to the potential profit for osteoporosis [21]. Clinical research shows that acupuncture is superior to Caltrate D in bone mineral density enhancement of postmenopausal osteoporosis [22]. Acupoint catgut embedding, a common kind of acupuncture, could increase the quality of life and adjust the reproductive endocrine and bone metabolism of postmenopausal women [23]. Acupuncture plus standard combination therapy for secondary osteoporosis in patient with spinal cord injury shows a better effect on the decreases of immunoglobulin M (IgM) and tumor necrosis factor *α* and the increase of bone mineral density in contrast with standard combination therapy only [8]. Hence, the qualitative and quantitative analysis of acupuncture for osteoporosis is vital.

### Objectives

The aim of this study is to review the efficacy and safety of acupuncture for osteoporosis.

## Methods

### Inclusive and exclusive criteria

#### Types of study

This review will be confined to randomized controlled trials (RCTs) comparing acupuncture with a control group. If the trial states the “randomization” phrase, it will be deemed a randomized study, and the blinding will not be restricted. The language will be limited as Chinese and English.

#### Types of participants

The participants of osteoporosis or osteopenia including postmenopausal osteoporosis, secondary or primary osteoporosis, and the fracture of different parts by osteoporosis will be included with no limitation of age, gender, and ethnic origin. Osteoporosis should be diagnosed clearly basing on bone mineral density with −2.5 < T score <−1.0 or T score ≤−2.5 detected by imaging device [24].

#### Types of interventions

Different types of stimulation (e.g., manual acupuncture, electro-acupuncture, transcutaneous electrical nerve stimulation, magnets, laser, acupoint catgut embedding, bleeding, acupoint injection, fire needle, needle knife, superficial needling, acupressure, cupping jar, or moxibustion) on specific points (e.g., traditional meridian points, Ashi points, auricular points, abdominal acupuncture points, and scalp acupuncture points) will be included.

The control interventions with drugs (conventional pharmaceutical medicine, herbs or herb extracts, no treatment or waiting list, placebo or sham, diet or physical activity therapy) will be included. Studies to compare the effect of different kinds of acupuncture stimulations or acupoints will be excluded. Besides, the comprehensive treatments containing acupuncture will be included if acupuncture could be tested by control group, such as acupuncture plus control vs. control.

### Types of outcome measures

#### Primary outcomes

1. Pain intensity
2. Bone mineral density
3. Global assessment of improvement proportion
4. Fracture
5. Mortality
6. Quality of life

#### Secondary outcomes

1. Biochemical indicators related to osteoporosis such as oestradiol (E2), serum calcium (Ca), phosphorus (P),bone Gla protein (BGP), alkaline phosphatase (ALP), calcitonin (CT), parathyroid hormone (PTH), interleukin-6 (IL-6), and others.
2. Side effects caused by acupuncture.

### Search methods for identification of studies

#### Electronic searches

A literature search will be conducted in the databases of Medline, Cochrane Library, Web of Science, EBASE, Springer, WHO International Clinical Trials Registry Platform (ICTRP), China National Knowledge Infrastructure (CNKI), Wanfang, Chinese Biomedical Literature Database (CBM), Chinese Scientific Journal Database (VIP) form the establishment to October, 2016.

#### Searching other resources

The other resources will be searched manually such as the references of all included studies of this systematic review and general review.

### Search strategy

The search strategy will be formulated in accordance with the guidance provided by the Cochrane Handbook. The Medline search strategy for title, abstract, and Medical Subject Heading (MeSH) is listed as Table 1, and others will be transformed basing on it.

**Table 1:** Medline search strategy for title, abstract and Mesh

Number	Search terms
1	randomised controlled trial
2	controlled clinical trial
3	randomly
4	randomized
5	randomised
6	trial
7	or/1-6
8	osteoporosis
9	osteopenia
10	bone mass loss
11	bone mass decrease
12	osteoporotic
13	or/8–12
14	acupuncture
15	acupoint
16	meridian
17	electro-acupuncture
18	electroacupuncture
19	transcutaneous electrical nerve stimulation
20	acupoint catgut embedding
21	acupressure
22	cupping jar
23	moxibustion
24	auricular points
25	abdominal acupuncture points
26	scalp acupuncture points
27	laser
28	magnets
29	Bleeding
30	acupoint injection
31	fire needle
32	needle-knife
33	superficial needling
34	or/14-33
35	7 and 13 and 34

### Data collection and analysis

#### Selection of studies

**Fig. 1:**
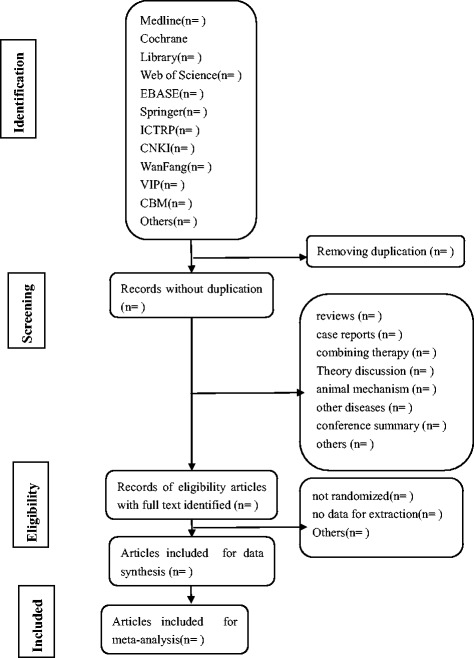
Flow diagram of studies identified

All the studied presupposed criteria will be selected and identified by the three authors (Taipin Guo, Xiao Chen, and Bowen Zhu). With the identification of title and abstract, the articles of duplicate and non-clinical trial will be expurgated. And then the intensive reading of full text could authenticate if some papers are ambiguous in title or abstract. The papers which do not meet the inclusion criteria will be excluded. Any disagreement should be resolved by discussion. The study of screening flow diagram is summarized as Fig. 1.

### Data extraction and management

The datum of each selected trial will be extracted and record in a special electronic form. The candidate information should be mainly composed of article general information (e.g., article identification, authors, publication year), study methods (e.g., randomization, allocation concealment, blinding, selective report, and other bias), participants (e.g., inclusion and exclusion criteria, demographic data like gender, age, duration of osteoporosis), details of invention in acupuncture and control groups, duration of treatment, outcomes, follow-up, side effect, and others. Two reviewers (Xiangnong Wu and Exian Shan) will complete the extraction independently and recheck crossly. Another reviewer (Yaju Jin) will literally inspect all the datum again. Bifurcations will be judged by an arbiter after discussion (Taipin Guo).

### Assessment of risk of bias in included studies

The bias risk of the following seven domains will be appraised by two reviewers (Xiantao Tai and Zili Liu) separately according to Cochrane handbook for systematic reviews of interventions [25]: generation of random sequences, allocation concealment, blinding, incomplete outcome data, selective reporting, and other issues. The items of low risk of bias, high risk of bias, or unclear risk of bias will be assigned to each issue. The Grading of Recommendations Assessment, Development and Evaluation (GRADE) will be used to evaluate the quality of each trial. The divergence will be resolved by discussion.

### Measures of treatment effect

Dichotomous data will be presented as risk ratio (RR) and 95 % confidence intervals (CI), while continuous outcomes will be showed as standard mean difference (SMD) 95 % CI.

### Unit of analysis issues

In order to avoid the unit of analysis error for multiple treatment groups in a trial, we will apply the approaches accordingly as follows: (1) separately reporting a pair wise comparison with more than one control group, (2) presenting subgroups of intervention result if there are two or more different stimuli kinds of acupuncture intervention groups, and (3) selecting and presenting the optimal group if there are two or more different acupoint prescription kinds of acupuncture intervention groups.

### Dealing with missing data

Referring to the Cochrane handbook for systematic reviews of intervention [25], we will try to contact the authors by telephone, email, or post to original investigators to request the missing data. If the missing data were not obtained, only the available data will be analyzed when it is assumed to be missing at random; otherwise, it will be imputed as the missing data with replacement values.

### Assessment of heterogeneity

The heterogeneity of the data will be calculated with *χ*^2^ test, and the values of *I*^2^ will be presented as the degree of heterogeneity. An interpretation of *I*^2^ is as follows: (1) 0 to 40 %: might not be important; (2) 30 to 60 %: may represent moderate heterogeneity; (3) 50 to 90 %: may represent substantial heterogeneity; and (4) 75 to 100 %: considerable heterogeneity.

### Assessment of reporting biases

The symmetry of funnel plots will be used to detect the reporting biases if more than 10 studies are included for meta-analysis.

### Data synthesis

All the primary and secondary outcomes will be merged, and meta-analysis using Cochrane Collaboration Review Manager software (RevMan V.5.3.5) will be performed. According to *Cochrane Handbook for Systematic Reviews of Interventions*, the fixed-effect model is used if the result may be viewed as a “typical intervention effect” from the studies included in the analysis, and the random-effects model is applied when the heterogeneity cannot readily be explained.

As some trails only report the outcomes as pre- and posttreatment values in intervention or control group, the mean change will be obtained by subtracting pre- from postmeasurements. Meanwhile, the standard deviation (s.d.) for changes will be calculated using the following formula: $\sqrt{\mathrm{s}.\mathrm{d}{.}^2\mathrm{p}\mathrm{r}\mathrm{e}+\mathrm{s}.\mathrm{d}{.}^2\mathrm{p}\mathrm{ost}-2\times r\mathrm{p}\mathrm{r}\mathrm{e},\mathrm{post}\times \mathrm{s}.\mathrm{d}.\mathrm{p}\mathrm{r}\mathrm{e}\times \mathrm{s}.\mathrm{d}.\mathrm{p}\mathrm{ost}},$ and the correlation coefficient (*r*_pre_, _post_) should be 0.5 [26]. Besides, the qualitative description will be implemented when the result is not fit to quantitative analysis.

### Subgroup analysis

We will conduct a subgroup analysis according to different types of stimulation (e.g., manual acupuncture, electro-acupuncture), such as subgroup 1 is manual acupuncture vs. drug, and subgroup 2 is electro-acupuncture vs. drug, and then to merge the subgroup results.

### Sensitivity analysis

According to the integrated results of heterogeneity and meta-analysis both in fixed-effect model and random-effects model, the sensitivity analysis will be carried out if there are great differences among the trials.

### Ethics and dissemination

This systematic review will not require data for individual patient and it does not relate to privacy issues. The results will be only disseminated on peer-reviewed publications and conference presentations.

## Discussion

Osteoporosis is a global prevalence disease, and it led to a great burden. There have no direct and efficient approaches to prevent and treat it by now. Acupuncture has been widely used to treat osteoporosis in China though lack of sufficient evidence. There has not any systematic review that has been reported by now. To synthesize the existing trials is vital, which could provide a new strong proof of the effectiveness and safety for clinicians, researchers, and health policy-makers.

The primary aim of this study is to test the effect of acupoint for osteoporosis. So, the different types of stimulation will be included either penetration of the skin or not. Meanwhile, different kinds of osteoporosis such as postmenopausal, senior, fracture will be incorporated. To explore and explain heterogeneity, subgroup analysis will be applied basing on the different kinds of acupuncture stimulation. This is the first time to do a systematic review about acupuncture for osteoporosis, and other outcomes reported in the original trials which may be omitted in this protocol will probably be included in the future.

## Abbreviations

DALY: Disability-adjusted life years
TENS: Transcutaneous electrical nerve stimulation
RANKL: Nuclear factor-kB ligand
TCM: Traditional Chinese medicine
IgM: Immunoglobulin M
RCT: Randomized controlled trials
E2: Oestradiol
Ca: Serum calcium
P: Phosphorus
BGP: Bone Gla protein
ALP: Alkaline phosphatase
CT: Calcitonin, PTH, parathyroid hormone
IL-6: Interleukin-6
ICTRP: International Clinical Trials Registry Platform
CNKI: China National Knowledge Infrastructure
CBM: Chinese Biomedical Literature Database
VIP: Chinese Scientific Journal Database
MeSH: Medical Subject Heading
SMD: Standard mean difference
CI: Confidence intervals
RR: Risk ratio
